# Supplementary material for: T-Cell Response against Varicella Zoster Virus in Patients with Multiple Sclerosis during Relapse and Remission
Source: Int J Mol Sci. 2021 Dec 28;23(1):298. doi: 10.3390/ijms23010298 (PMC8745673; doi:10.3390/ijms23010298)
Supplement: Supplementary file 1 [file ijms-23-00298-s001.zip › ijms-1418986-supplementary.pdf]

Supplementary Table S1. Clinical, demographic and T cell proliferation data of MS patients

Clinical data							Proliferation (%)											
							CD4+				CD8+				Treg			
							VZV		AV or EBV*		VZV		AV or EBV*		VZV		AV or EBV*	
Patient	Sex/ Age (years)	Evolution time (years)	Relapses/ year	EDSS	Current Treatment	Days of Relapse	Rel	Rem	Rel	Rem	Rel	Rem	Rel	Rem	Rel	Rem	Rel	Rem
1	F/28	1.5	3	3	Teriflunomide	2	0	1.4	0.0	0.3	0.9	0.3	0.0	0.0	50.0	0.0	12.5	0.0
2	F/26	7	2	1.5	None	2	0	1.6	0.3	1.2	4.7	2.3	0.9	1.0	25.0	0.0	25.0	2.7
3	M/37	3	1	4	IFN	2	0.0	-	0.0	-	0.0	-	0.0	-	0.0	-	0.0	-
4	F/21	3	3	6.5	IFN	4	8.4	-	6.6	-	16.2	-	10.3	-	16.7	-	0.0	-
5	M/27	3	2	2	IFN	7	1.8	5.5	0.0	4.4	2.0	9.5	3.3	5.5	0.0	0.0	0.0	0.0
6	F/42	5	1	5	None	2	6.9	6.4	4.5	4.3	1.6	12.1	0.0	9.3	0.0	5.4	0.0	5.6
7	M/33	11	2	6.5	Azathioprine	7	3.7	0.0	3.9	0.0	19.6	0.0	0.3	0.0	0.0	0.0	0.0	12.5
8	F/34	4	2	1	Azathioprine	8	7.7	0.0	0.0	2.5	5.6	0.0	0.0	6.2	5.9	0.0	0.0	0.0
9	F/31	2	0.5	3	None	7	3.7	-	3.4	-	8.6	-	6.9	-	0.0	-	8.4	-
10	M/37	10	1	6.5	Methotrexate and carbamazepine	5	0.0	3.3	0.0	2.4	0.0	6.4	2.2	5.0	0.0	5.9	0.0	0.0
11	F/26	0.33	NA	1	None	7	6.9	6.7	0.0	2.5	3.9	8.2	0.7	7.9	0.0	0.0	0.0	0.0
12	F/30	3	1	1.5	IFN	8	2.3	0.0	4.0	0.0	0.0	0.0	0.0	0.0	21.4	0.0	14.3	0.0
13	M/31	2	0.5	5.5	Teriflunomide	GD	0.0	23.3	0.0	9.9	0.0	19.7	0.0	10.1	0.0	0.0	0.0	0.0
14	F/37	12	1	2	None	5	0.0	2.8	0.1	2.6	0.0	6.4	2.9	3.3	11.1	6.7	40.0	0.0
15	F/33	0.08	NA	1	None	1	8.3	-	4.4	-	20.9	-	4.2	-	0.0	-	0.0	-
16	F/22	4	1	2	None	2	2.0	-	0.6	-	10.6	-	0.8	-	13.3	-	33.3	-
17	F/30	3	2	2	None	8	3.6	0.0	2.5	0.0	21.7	0.0	0.0	2.2	0.0	0.0	0.0	5.9
18	M/20	1.5	3	4	None	8	0.4	-	0.5	-	0.0	-	0.4	-	0.0	-	0.0	-
19	M/25	8	2	5.5	None	8	1.1	19.0	0.1	17.9	0.0	19.6	0.0	4.6	6.7	0.0	0.0	0.0
20	F/26	0.6	3	3.5	MTP	4	0.0	1.9	0.2	1.3	0.0	1.6	0.7	0.9	0.0	7.7	0.0	3.1
21	M/25	3	1	1	MTP	5	0.0	-	0.0	-	0.7	-	1.0	-	0.0	-	0.0	-
22	F/31	16	2	4	None	4	0.2	2.6	0.3	3.2	0.1	5.7	1.9	6.8	0.0	0.0	0.0	0.0
23	M/25	3	0.5	1.5	None	2	1.1	1.2	0.0	1.0	2.5	1.0	0.7	0.0	0.0	0.0	12.5	0.0
24	F/29	3	1	6	None	GD	1.9	2.0	7.0	1.8	14.7	8.0	12.3	8.0	0.0	0.0	16.7	0.0
25	F/39	14	0.2	1	IFN	3	1.2	2.8	2.0	0.5	0.8	8.4	6.8	0.0	0.0	9.1	0.0	15.0
26	M/23	2	3	3	Natalizumab	3	8.0	3.3	3.2	1.1	3.0	6.0	0.0	2.3	0.0	11.8	6.3	33.3

27	F/34	7	3	2	Glatiramer acetate	3	0.0	3.6	0.0	3.4	0.0	5.2	0.0	4.6	0.0	11.7	0.0	3.3
28	F/52	27	1	7	None	8	2.3	0.2	0.4	0.0	1.7	0.0	0.8	0.2	0.0	0.0	0.0	0.5
29	M/17	8	0.5	6.5	Azathioprine	1	0.0	5.6	0.0	3.3	5.2	3.6	0.0	8.3	0.0	0.0	16.7	0.0

*Cells from patients 1 to 19 were stimulated with AV, while cells from patients 20 to 29 were stimulated with EBV

NA: Not applicable

GD: new gadolinium-enhanced lesion without clinical manifestation

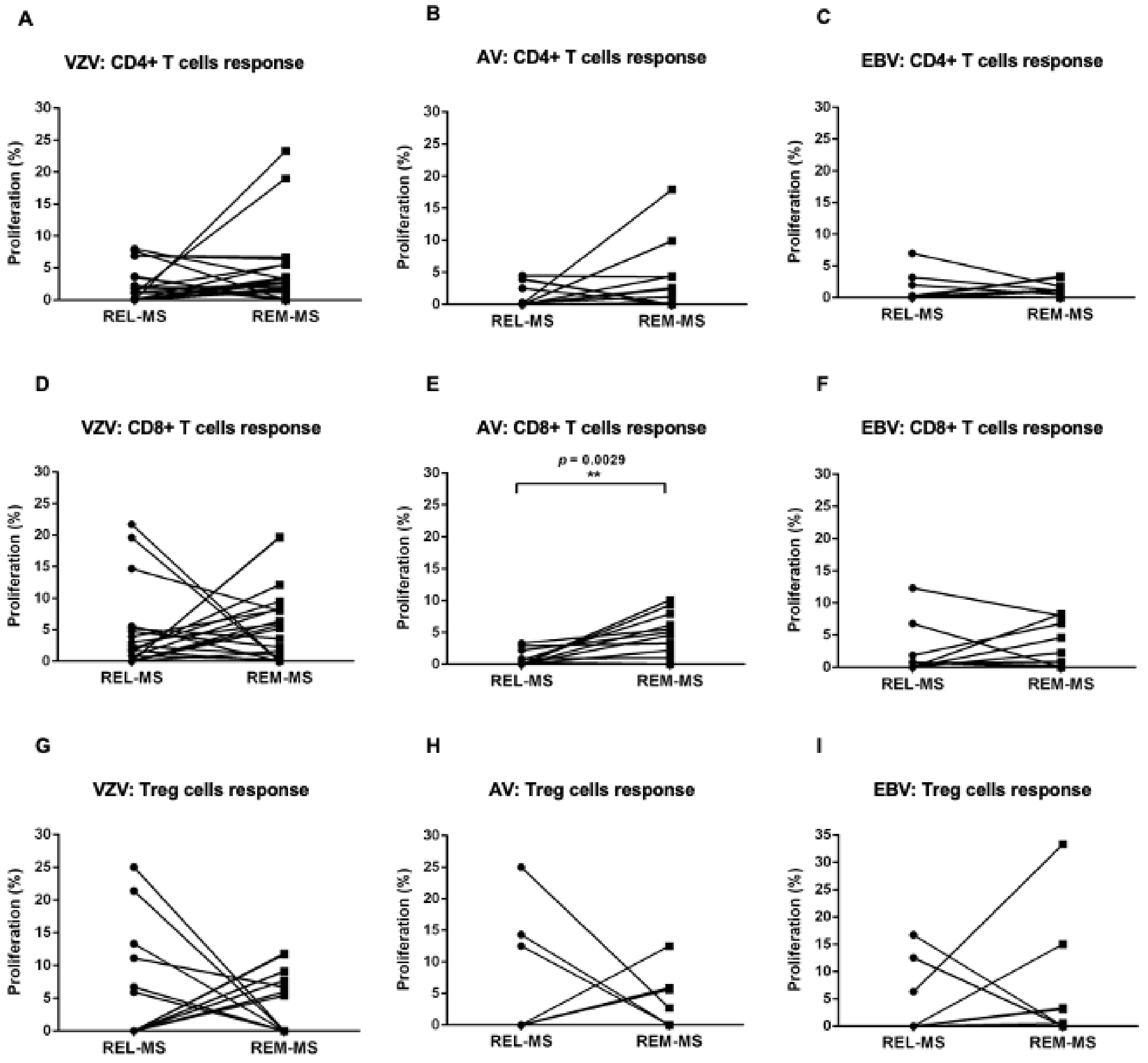

Supplementary Figure S1. Paired Wilcoxon tests of relapse and remission cells from each subject (CD4+, CD8+ and Treg cells) to stimulation with VZV, AV or EBV. Comparison of the T cell response to VZV (n = 22), AV (n = 13) and EBV (n = 9) between paired REL-MS and REM-MS samples show higher proliferation trends of effector CD4+ and CD8+ T lymphocytes during remission, with statistical significance only in CD8+ T cells when stimulated with AV (**A-F**). For Treg cells, there was no difference between relapse and remission status. (**G-I**). Each dot represents one subject. $^{**}p < 0.01$.
